# IL-18 associated with lung lymphoid aggregates drives IFNγ production in severe COPD

**DOI:** 10.1186/s12931-017-0641-7

**Published:** 2017-08-22

**Authors:** Emmanuel Briend, G. John Ferguson, Michiko Mori, Gautam Damera, Katherine Stephenson, Natasha A. Karp, Sanjay Sethi, Christine K. Ward, Matthew A. Sleeman, Jonas S. Erjefält, Donna K. Finch

**Affiliations:** 1 MedImmune Ltd, Granta Park, Cambridge, CB21 6GH UK; 2 MedImmune LLC, 1 MedImmune Way, Gaithersburg, MD USA; 3 Department of Experimental Medical Science, BMC D12, Lund University, SE-221 84 Lund, Sweden; 4 Department of Respiratory Medicine and Allergology, Lund University Hospital, Lund, Sweden; 5 Department of Medicine, University at Buffalo, 3495 Bailey Avenue, Buffalo, NY 14215 USA; 6 Quantitative Biology IMED, AstraZeneca R&D, Cambridge, UK; 7 Present address: Agenus Ltd, Cambridge, UK; 8 Present address: University of Nottingham, Nottingham, UK; 9 Present address: Bristol-Myers Squibb, Princeton, NJ USA; 10 Present address: Regeneron Pharmaceuticals Inc, Tarrytown, NY USA

**Keywords:** Interleukin-18, Interferon gamma, Tertiary follicles, Lymphoid aggregates, Lymphocytes, Chronic obstructive pulmonary disease

## Abstract

**Background:**

Increased interferon gamma (IFNγ) release occurs in Chronic Obstructive Pulmonary Disease (COPD) lungs. IFNγ supports optimal viral clearance, but if dysregulated could increase lung tissue destruction.

**Methods:**

The present study investigates which mediators most closely correlate with IFNγ in sputum in stable and exacerbating disease, and seeks to shed light on the spatial requirements for innate production of IFNγ, as reported in mouse lymph nodes, to observe whether such microenvironmental cellular organisation is relevant to IFNγ production in COPD lung.

**Results:**

We show tertiary follicle formation in severe disease alters the dominant mechanistic drivers of IFNγ production, because cells producing interleukin-18, a key regulator of IFNγ, are highly associated with such structures. Interleukin-1 family cytokines correlated with IFNγ in COPD sputum. We observed that the primary source of IL-18 in COPD lungs was myeloid cells within lymphoid aggregates and IL-18 was increased in severe disease. IL-18 released from infected epithelium or from activated myeloid cells, was more dominant in driving IFNγ when IL-18-producing and responder cells were in close proximity.

**Conclusions:**

Unlike tight regulation to control infection spread in lymphoid organs, this local interface between IL-18-expressing and responder cell is increasingly supported in lung as disease progresses, increasing its potential to increase tissue damage via IFNγ.

**Electronic supplementary material:**

The online version of this article (doi:10.1186/s12931-017-0641-7) contains supplementary material, which is available to authorized users.

## Background

The pathogenesis of Chronic Obstructive Pulmonary Disease (COPD) is associated with epithelial dysfunction and the excessive recruitment of inflammatory cells in response to chronic insult of the lung [1]. Among tissue infiltrating leukocytes are effector cells of both innate and adaptive immune responses, such as neutrophils, macrophages, NK cells, T cells and B cells [2]. Apart from classic tissue infiltration, accumulation of immune cells in COPD lungs is also manifested as formation of bronchial/bronchiolar-associated as well as alveolar lymphoid aggregates (LA), and the number of these structures increases with disease severity [2, 3]. This de novo formation of lymphocyte and macrophage-rich lymphoid aggregates, referred to as ectopic lymphoid tissue, is a well-recognised example of immunological remodelling in COPD. Whilst intact immune responses to pathogens are required to protect vulnerable COPD patients from infection, an inappropriate response in a chronically inflamed lung may trigger exacerbations of symptoms and contribute to accelerated decline in lung function [4].

Underlying mechanisms responsible for lung tissue destruction are not well understood but an aberrant response of NK cells and T cells, CD8^+^ T cells in particular, is thought to play an important role [5, 6]. The dysregulation of the cytotoxic function of NK cells and CD8^+^ T cells may lead to the destruction of the lung parenchyma through excessive response to pathogens and bystander damage to surrounding tissues. Production of interferon gamma (IFNγ) by inflammatory T cells, NK and NKT cells is an important component of the host response, inhibiting viral replication, promoting and perpetuating antigen presentation via increased expression of MHC molecules and priming other leukocytes, notably macrophages [7]. In lymph nodes that drain infected tissues, prompt lymphocyte-macrophage communication, involving rapid production of IFNγ from resident lymphocytes and release of interleukin-18 from proximally associated macrophages, is crucial to limit the spread of infection [8]. This response is too rapid to be explained by conventional adaptive T cell function or plasticity. Kastenmuller et al. [8] demonstrated the critical role of IL-18, and also an additional unidentified second signal, in this innate IFNγ response, and established the requirement for spacial organisation of the macrophages and lymphocytes within the lymph node to facilitate it.

There is ample clinical evidence of IFNγ being a significant component of the immunopathology in COPD. IFNγ is increased in the airways of COPD patients [9, 10] and associated with viral exacerbations of disease [11, 12]. Furthermore, IFNγ-positive T cells are increased in COPD patients [13] where disease severity correlates with IFNγ release by peripheral patient-derived CD8^+^ cells [14]. The fact that excessive IFNγ in the lungs of mice is associated with development of airspace enlargement [15, 16] further underscores the pathogenic potential of IFNγ. Therefore, better understanding of the cellular and molecular regulation of IFNγ in the lungs of COPD patients is an essential step towards determining whether this mechanism plays a part in the link between excessive chronic inflammation, exacerbation and increase in the rate of decline.

We and others showed previously that IL-1α, IL-1β and IL-18 were increased in the lung of COPD patients [17, 18] and that these mediators were released by epithelial cells on infection with rhinovirus [19]. Consequently, we hypothesised that these cytokines could chronically stimulate an innate IFNγ response via local lymphocyte-macrophage communication in inflamed lungs as well as during exacerbation of disease. In this regard, ectopic lymphoid follicle formation in COPD is of particular interest. The present study investigates which mediators most closely correlate with IFNγ in sputum in stable and exacerbating disease, and seeks to shed light on the spatial requirements for innate production of IFNγ, as reported in mouse lymph nodes, to observe whether such microenvironmental cellular organisation [8] is relevant to IFNγ production in COPD lung.

## Methods

### COPD sputum samples: COPD cohort 1

After protocol approval (Veterans Affairs Western New York Healthcare System (Buffalo, NY)) and after obtaining written informed consent, COPD patients with chronic bronchitis were recruited in a prospective longitudinal study which has been previously reported ([20], Additional file 1: Table S1). From 1994, spontaneously produced sputum samples were collected monthly when patients were clinically stable, as well as whenever patients were suspected of an exacerbation before specific treatment, and on convalescence from each exacerbation episode. Sputum samples were homogenized with dithiothreitol, aliquots removed for bacterial cultures and the rest was centrifuged to obtain sputum pellets and supernatants as previously described [20]. Triplets of sputum supernatants obtained before, during and after an exacerbation for 35 episodes occurring in 24 patients were selected for this study (7 patients had 2 exacerbations, and 2 patients had 3 exacerbations in the course of the study).The concentration of 175 analytes was measured using human MAPv1.6 multiplex assay (Myriad RBM Inc., Austin, TX, USA).

The least detectable dose (LDD) was determined by adding three standard deviations to the average of the signal for 20 replicate determinations of the standard curve blank. The LDD for the analytes of interest were 7.11 pg/ml for IL-1α, 1.1 pg/ml for IL-1β, 25 pg/ml for IL-18, 3.2 pg/ml for IFNγ, 19 pg/ml for IL-2, 59 pg/ml for IL-7, 26 pg/ml for IL-12, 580 pg/ml for IL-15, 30 pg/ml for IP-10, 100 pg/ml for MIG, 34 pg/ml for ITAC and 0.8175 pg/ml for RANTES. Where values were returned from the assay that were below the least detectable dose they were included in the statistical analysis as a best estimate, rather than return missing values.

### Immunohistochemistry: COPD cohort 2

Matched tissue samples for immunohistochemistry were not available from COPD cohort 1 (above) in which sputum analysis was performed. Paraffin sections (4 μm) were generated from surgical lung samples from 31 COPD subjects, eight never-smokers and seven smokers with normal lung function (Additional file 1: Table S2) from a patient cohort in which, after appropriate and usual patient care, optimal tissue samples were acquired for immunohistochemistry. The study was approved by the local ethics committee (Lund, Sweden). Sections were subjected to high pH heat-induced epitope retrieval and after a blocking step, IL-18 immunostaining was performed using a rabbit polyclonal antibody (HPA003980, Sigma-Aldrich, USA) followed by detection using a polymer consisting of a dextran core with attached secondary anti-rabbit antibodies and horseradish peroxidase (Dako Envision Detection System, Glostrup, Denmark). Finally, a brown colored immunoreaction product was produced using the peroxidase substrate diaminobenzidine (DAB, Dako) as chromogen. Sections were counterstained with Mayer’s haematoxylin. Anti-IL-18 antibody specificity was validated by pre-absorption tests with recombinant IL-18 protein.

IL18^+^ and IL-18^−^ cells were identified by double immuno-fluorescence staining with antibodies against CD68 (Dako), CD163 (Novocastra, Bromma, Sweden), CD11c (Dako), CD1a (Novocastra), Langerin (Novocastra), CD21 (Novocastra), CD20 (Dako), CD138 (Dako), CD3 (Dako), CD56 (Novocastra), ECP (Diagnostics Development, Uppsala, Sweden), MPO (Dako) and tryptase (Chemicon, Solna, Sweden). Briefly, the primary anti-IL-18 antibody was detected by a biotinylated goat anti-rabbit antibody (Vector Laboratories, Peterborough, UK) followed by fluorochrome-labelled streptavidin (Alexa 555, Invitrogen). Next, the antibody against the cell marker of interest was applied and detected by a fluorochrome-labelled secondary antibody (Alexa 488, Invitrogen). As background staining, cell nuclei were visualized by the DNA–binding fluorochrome (Hoechst 33,342, blue nuclei). In some instances, IL-18^+^ immunoreactivity was visualized using Vina green chromogen (Biocare Medical, Concord, CA, USA) and NK cells were co-stained using an anti-CD56 antibody (Novocastra) visualized with brown DAB chromogen (Dako).

Lymphoid aggregates (defined as clusters of >50 lymphoid cells) were quantified on high resolution digital images of whole tissue sections using computerized image analysis (Scanscope slide scanner/ImageScope, Aperio Technologies). The percentage of the epithelial circumference with a distinct apical IL-18 staining was calculated by manually outlining the epithelium and computerized image analysis.

### Infection of epithelial cells with human rhinovirus

Human Rhinovirus 14 (HRV14) obtained from the American Type Culture Collection (LGC Standards, UK) was prepared as described previously [19]. Normal Human Bronchial Epithelial cells (NHBE, Lonza, Switzerland) were seeded at 3.2 × 10^4^/cm^2^ in supplemented bronchial epithelial basal medium (Lonza). The following day the cells were infected with HRV14 (MOI 0.007) for 3 h. The medium was exchanged and the supernatant was collected after 48 h for cytokine analysis or transfer experiments.

### Stimulation of monocytes

Human peripheral blood mononuclear cells (PBMC) were isolated from leukocyte cones (supplied by NHS Blood and Transplant Service (NHSBT, UK) as anonymized samples from consenting donors) by layering over Ficoll-Paque Plus per manufacturer’s instructions (GE Healthcare, UK). Monocytes were obtained by negative selection (Stem Cell Technologies, France). For the stimulation, 5 × 10^4^ monocytes were treated with 1 ng/mL LPS (Sigma, UK) for 24 h before the supernatant was collected for transfer experiments or cytokine analysis.

### Induction of IFNγ secretion by PBMC and NK cells

Human PBMC (2 × 10^5^ cells/well) were stimulated with 1 ng/ml of IL-12 (R&D Systems) and 1 ng/ml of LPS in the presence of an IL-1R antagonist at 100 nM (anakinra, Amgen/Biovitrium), an IL-18 antagonist at 33 nM (IL-18BPa-Fc, R&D Systems) or a control IgG1 at 33 nM (MedImmune, UK). Alternatively, NK cells were isolated from PBMC by negative selection (Stem Cell Technologies) and plated (1 × 10^4^ cells/well) in medium containing 1 ng/ml IL-12. Supernatants taken from HRV-activated NHBE cells or LPS-activated monocytes were mixed with antagonists indicated above and then added to NK cells at a dilution of 1:4. For transwell and co-culture experiments, 5 × 10^4^ NK cells were seeded either in the well along with 5 × 10^4^ monocytes (co-culture) or in an insert (transwell). Supernatants were collected after 24 h of incubation to measure IFNγ production.

### Detection of IL-1, IL-18 and IFNγ in the supernatant of stimulated cells

The amount of IL-1α and IL-1β was determined using a Duoset ELISA kit (R&D Systems, Abingdon, UK) following manufacturer’s instructions except for the detection system (see below). The production of IL-18 was measured using a commercial antibody pair consisting of clone 125–25 as a capture antibody and biotinylated clone 159-12B as a detection antibody (R&D Systems). Similarly, IFNγ concentration was determined using a sandwich ELISA made of clone NIB42 as capture antibody and biotin-conjugated clone 4S.B3 as a detection antibody (BD Biosciences, Oxford, UK).

In all these ELISA, the final detection step was based on a dissociation-enhanced time resolved fluorescence method using Europium-labelled streptavidin as recommended by the manufacturer (DELFIA system, Perkin Elmer, Boston, MA, USA).

### Statistical analysis

**Fig. 1:**
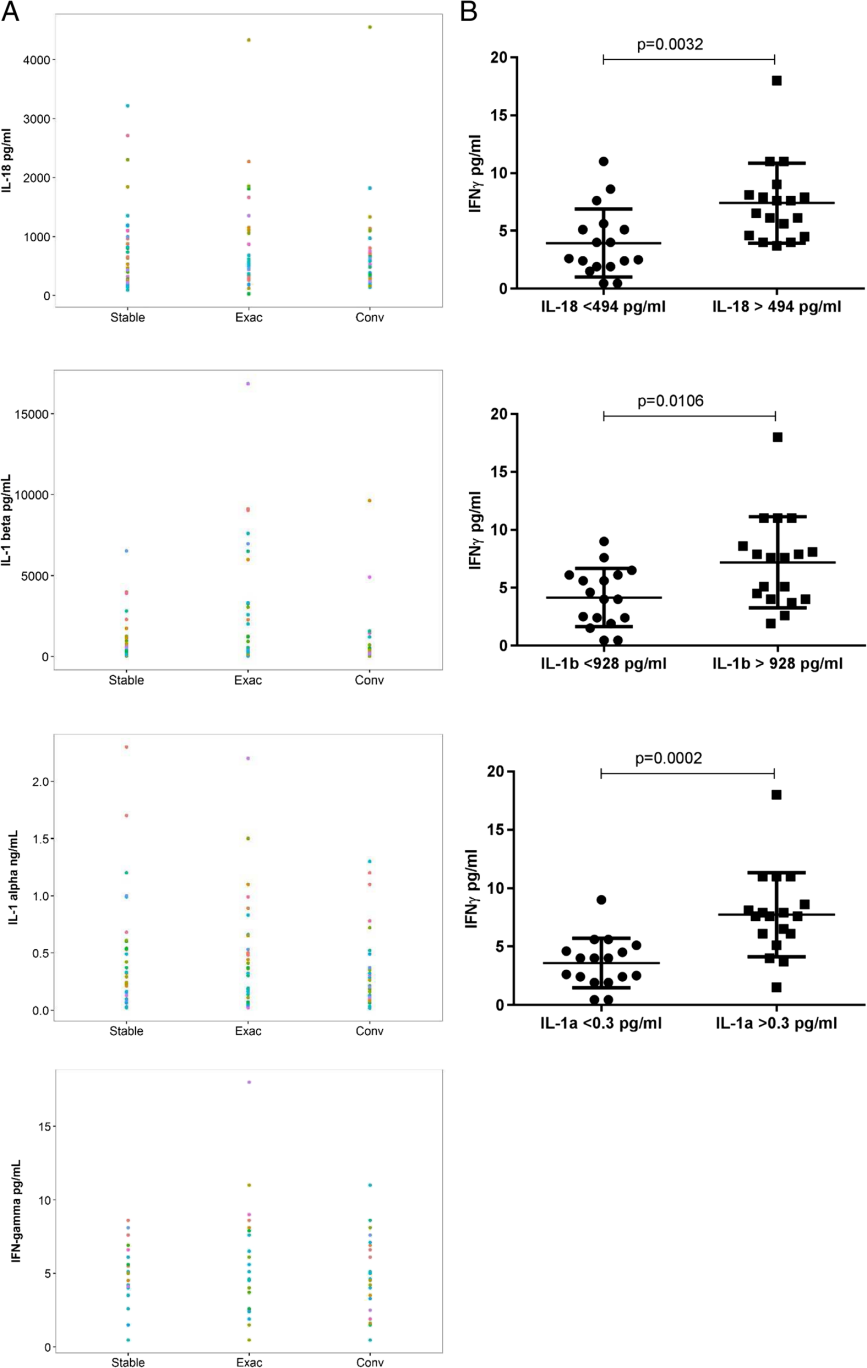
High levels of sputum IFNγ are associated with high levels of IL-18, IL-1α or IL-1β. **a** The concentrations of IL-18, IL-1α and IL1β in 35 sputum samples from patients either stable, having an exacerbation or convalescent were determined using the human MAPv1.6 multiplex assay. Results are colored by patient ID, and the data were fitted with a linear mixed effects model to account for patient variability and test for significant difference in exacerbation compared to stable or convalescence samples (see Additional file 1: Table S3). **b** Patients were grouped according to the median value at exacerbation of IL-18, IL-1α and IL-1β and the corresponding sputum IFNγ values were plotted. Lines indicate median. Statistical significance was assessed using a Student’s t-Test.

See figure legends and table footnotes for statistical information. Non-parametric methods were utilised where data were not normally distributed. The experimental unit through the manuscript is the individual patient. Where multiple technical readings were obtained for a measure these were averaged. Analysis of the sputum analyte data displayed in Fig. 1a took into account the longitudinal nature of the study and paired patient samples, as follows. A linear mixed effect model (Eq. 1) was fitted to each variable of interest to assess the impact of status (convalescence, stable or exacerbation) on the variable and account for the repeat nature of the data by fitting PatientID as a random effect.

1 $$ \mathrm{Y}\sim \mathrm{Status}+\left(1|\mathrm{PatientID}\right) $$

**Fig. 2:**
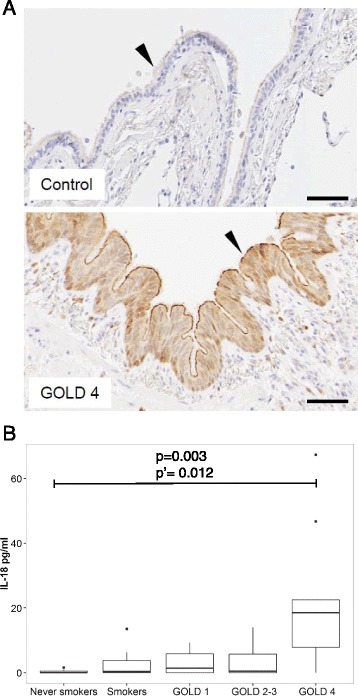
IL-18 is expressed by lung epithelial cells and is associated with an apical lining in GOLD 4 COPD patients. **a** Bright field micrographs exemplifying epithelial IL-18 immunostaining in a control lung sections (upper panel) and in GOLD 4 patients (lower panel). Immunoreactivity is visualized by brown DAB chromogen. Arrow head indicates apical surface of small airway. Scale bar = 70 μm. **b** Quantification of IL-18 apical staining across patient groups. Each data point represents a mean value for multiple airways analysed for each patient; Number of patients in each group: Never smokers *n* = 8, Smokers *n* = 7, COPD GOLD Stage 1 *n* = 6, COPD GOLD Stage 2–3 *n* = 14 and COPD GOLD Stage 4 *n* = 10. Numbers of airways quantified for each patient varied but were >3 per patient. Data is summarised with a boxplot, showing the minimum, maximum, first quartile, third quartile and median. Outliers are identified as data points beyond the first and third quartile by at least the 1.5xInterquartile range. A generalised least square model with heterogeneous variance was fitted to explore the effect of the smoking/disease status on the IL-18 readings

A likelihood ratio test was used to assess the covariance structure for the residuals and if significant (*p* < 0.05) a heterogeneous variance model was fitted. A Q-Q plot of residuals and a plot of residuals against predicted assessed the model fit. The stable status was set as the reference and the alternates were tested for their contribution to the model using an F-test. To manage the multiple testing, within a model a Bonferroni adjusted threshold was used to control the family wise error rate to 5%. For the analysis of data displayed in Fig. 2b, a generalised least square model was fitted to explore the effect of the smoking/disease status on the IL-18 readings. Variance was found to depend on status and so a heterogeneous variance model was fitted. A Q-Q plot of residuals and a plot of residuals against predicted, coloured by various factors, assessed the model fit. The Never Smoking status was set as the reference and the alternates were tested for their contribution to the model using an F-test. To manage the multiple testing, within a model a Bonferroni adjusted threshold was used to control the family wise error rate to 5%.

## Results

### COPD sputum IFNγ correlates with IL-1α, IL-1β and IL-18

To identify pro-inflammatory soluble mediators that might be involved in promoting IFNγ production locally in the lung, we determined sputum concentrations of 175 analytes in longitudinal sputum samples from 24 patients with COPD (patient demographics in Additional file 1: Table S1). IFNγ was detected above the least detectable dose (LDD) of the assay in 82 out of 105 samples. IL-18 was among the top 11 analytes most significantly correlated with IFNγ and with a Pearson’s rank *r*_*s*_ > 0.5 (Table 1), together with IL-1α and IL-1β that belong to the same cytokine family. Among other molecules which correlated closely with IFNγ were molecules considered markers of increased inflammation in the lung, such as C-reactive protein, fibrinogen, haptaglobin and α1 anti-trypsin. Conversely, other cytokines associated with T cell responses and IFNγ production such as IL-12p70, IL-15, IL-2, IL-7 were only detectable in a very small number of samples. IFNγ did not correlate with T cell chemotactic factors such as RANTES, IP-10, MIG and ITAC.

**Table 1:** Analytes most significantly associated with IFNγ in COPD sputum samples

Analyte	Median (range)	P′-value	r_s_
IFNγ (pg/ml)	5.05 (18–0.45)	-	1
IL-1 receptor antagonist (ng/ml)	202 (16.7–507.3)	2.91E-18	0.72
IL-1α (pg/ml)	225 (17–2290)	1.56E-14	0.66
C-Reactive Protein (ng/ml)	0.035 (0.0132–150)	6.31E-11	0.58
sEGFR (ng/ml)	4.584 (0.385–48.064)	6.87E-11	0.58
α1-Antitrypsin (μg/ml)	11.2 (1.25–140)	1.05E-10	0.58
Haptoglobin (ng/ml)	4.26 (0.0177–62.2)	1.95E-10	0.57
Fibrinogen (ng/ml)	0.905 (0.0569–2.59)	1.98E-10	0.57
IL-1β (pg/ml)	386 (7.39–16,850)	6.57E-10	0.56
IL-18 (pg/ml)	513 (22.1–4550)	1.23E-09	0.55
α2-Macroglobulin (μg/ml)	14.5 (0.915–228)	2.31E-09	0.54

The concentration of 175 analytes was determined in 105 sputum samples collected pre-, during and post-exacerbation of COPD in 24 patients using the human MAPv1.6 (Myriad RBM Inc).The top 11 analytes that had a significant correlation with IFNγ are shown in the table. Correlation was assessed by Spearman rank order correlation (r_s_) irrespective of whether stable, exacerbation or convalescence sample. P′-value is the significance value adjusted for multiple testing using Hochberg method to control the family wise error rate to 5%

Since infections often are associated with exacerbation of COPD symptoms we looked at the modulation of IFNγ, and the 10 analytes which correlated most closely with it in sputum either pre-, during or post-exacerbation (Fig. 1a, Additional file 2: Figure S1; Additional file 1: Table S3). For all 11 parameters, there were no statistical significant difference between stable and convalescence levels. IL-1β, α2-macroglobulin, α-1 anti-trypsin, and haptoglobin had a statistically significant increase when the status was exacerbation compared to stable. We also analysed variance in the data. For IFNγ, IL-1β, IL-1α, α2-macroglobulin, α1 anti-trypsin, haptoglobin, and C- reactive protein the variance was significantly higher for the exacerbated state compared to the stable condition, and in some cases was also higher in convalescence samples compared to baseline. For example, the variance in anti-trypsin was ~10 fold higher in the convalescence and exacerbated state compared to the stable state (Additional file 1: Table S3) IFNγ was found to correlate with IL-1α, IL-1β and IL-18 irrespective of exacerbation or baseline/convalescence, with Spearman’s rank *r*_*s*_ values marginally higher during acute exacerbation of COPD (Additional file 2: Figure S1). Stratifying patients according to the median value of IL-1α, IL-1β or IL-18, clearly demonstrated that a high level of these three cytokines was associated with significantly higher level of IFNγ (Fig. 1b). IFNγ, IL-1α, IL-1β and IL-18 sputum levels did not correlate with severity of disease as measured by FEV1, FEV1/FVC or FEV1% predicted in this cohort although the sample size was likely too small for this analysis to be meaningful (*p* > 0.05; data not shown).

### Lymphoid aggregate-associated macrophages and dendritic cells are a primary source of IL**-**18 in COPD lungs

Previously we reported the expression of IL-1α and IL-1β in COPD lungs and their association with neutrophilia [17]. Interleukin-1α was found to be associated primarily with inflammatory macrophages, while IL-1β was expressed by macrophages and epithelial cells. To characterise the source of lung IL-18 in this study and investigate its possible dysregulation in COPD, we carried out a detailed mapping of the IL-18 localization by immunohistochemistry using tissue sections from COPD patients of different disease severity (*n* = 31), never-smokers (*n* = 8) and smokers with normal lung function (*n* = 7). Patchy IL-18 immunoreactivity of variable intensity was found in epithelial cells in small airways both in controls and COPD patients (Fig. 2a). There was also a noticeable association of IL-18 staining with the apical border of epithelial cells. Quantification of the epithelial staining across the different GOLD stages showed a statistically significant increase in IL-18 apical border staining in patients with GOLD 4 severity (Fig. 2b). There was no statistically significant difference in the IL-18 levels for Smokers, GOLD 1 nor GOLD 2–3 patient samples relative to Non-Smokers. However, GOLD 4 patient samples had statistically significant elevated epithelial staining (*p* = 0.003, adjusted for multiple testing p’ = 0.012) where the readings were higher by 21.05 ± 6.7 (estimate ± standard error). The variance was found to be heterogeneous (*p* < 0.0001) and this arose as the variance being higher relative to the Non-Smokers in the Smoker, GOLD 1 and GOLD 2–3 group by an average of 8 fold and the variance in the GOLD 4 group being 35.8 fold higher.

**Fig. 3:**
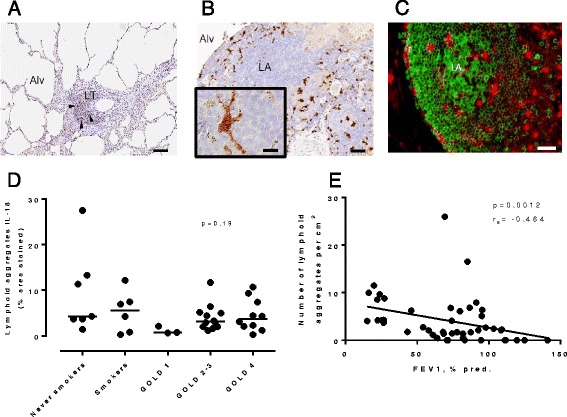
Strong IL-18 staining associated with dendritic-shaped cells in lung lymphoid aggregates in COPD lung sections. **a**-**b** Bright field micrographs exemplifying lymphoid tissue-associated IL-18 immunoreactivity (brown) in COPD lung sections containing (**a**) a small lymphoid aggregate (LA) and (**b**) a large well-developed lymphoid aggregate in GOLD 4 COPD. Scale bars: A = 100 μm; B = 35 μm. Alv alveolar space; LA Lymphoid aggregate; LT tertiary lymphoid structure; arrow heads identify strongly positive IL-18 cells. **c** Double immunofluorescence staining for CD20 (green Alexa-488 fluorophore) identifying B-cell aggregates and IL-18 positive cells (red Alexa-555). Scale bar = 30 μm. **d** Within lymphoid aggregates, the density of IL-18 positive cells does not change across study groups as determined by the quantification of the area of IL-18 positive cells (Kruskall-Wallis test *p* = 0.19 not significant). Bar indicates the median value seen within a group. **e** The inverse correlation (Spearman r_s_ = −0.46, *p* = 0.0012) of the number of lymphoid aggregates with FEV1 (% predicted) across study groups indicates that the overall number of IL-18 positive cells in the lung parenchyma increases with disease severity

Regardless of the group, the strongest IL-18 signal was associated with cells with a dendritic-like shape that were identified as CD68^+^ CD163^+^ macrophages and CD68^−^ CD163^−^ CD11c^+^ dendritic cells. These IL-18^+^ myeloid cells were most commonly observed in lymphoid aggregates, notably in perifollicular regions (Fig. 3a-c **,** Table 2). Interestingly, well-developed larger lymphoid aggregates, which were most commonly found in severe disease, also contained large germinal center-associated IL18^+^, CD68^+^ but CD163-negative macrophages with a dendritic morphology. A similar pattern of staining could be observed in lung-draining lymph nodes (Additional file 2: Figure S2). The area of IL-18^+^ immunoreactivity within each individual lymphoid aggregate was similar among the study groups (Fig. 3d **;** Kruskall Wallis test *p* = 0.19 not significant), however the total number of IL-18^+^ cells in the lung tissue overall was found to be increased in subjects with GOLD stage 4 COPD compared to controls because the total number of lymphoid structures in the lung tissue was inversely correlated to FEV_1_ as assessed by Spearman rank order correlation (Fig. 3e; r_s_ = −0.464, *p* = 0.0012). In addition, IL-18^+^ subcapsular-like cells could be observed in large follicles in the lung (Fig. 3b, c); similar to what we observed in lung draining lymph nodes (Additional file 2: Figure S2 I-J) and to what has been described in secondary lymphoid organs in mice [8]. Outside lymphoid structures, a modest IL-18 staining was found in alveolar macrophages, subepithelial bronchiolar macrophages and pulmonary vessel macrophages (Table 2). No IL-18 staining was found to be associated with ECP^+^ eosinophils, MPO^+^ neutrophils, tryptase^+^ mast cells, CD3^+^ T cells, CD20^+^ B cells or CD138^+^ plasma cells. The strong spatial association of IL-18 with lymphoid aggregates in the lung of COPD patients is thus consistent with a role in promoting NK and T cell responses.

**Table 2:** IL-18 immunoreactivity of lung macrophages and dendritic cells

Cell type	Markers	Relative IL-18 positivity
Macrophage populations		
Small airway Mac	CD68^+^, CD163^+^	+
Alveolar, interstitial Mac	CD68^+^, CD163^+^	+
Alveolar, luminal Mac	CD68^+^, CD163^+^	+(+)
Pulmonary vessel Mac	CD68^+^, CD163^+^	(+)
Lymphoid aggregates Mac	CD68^+^, CD163^+^	+++
Dendritic cell populations		
Mucosal DCs	CD1a^+^, Langerin^+^	−
Small airway mDCs	CD68^−^, CD163^−^, CD11c^+^	+
Lymphoid aggregates mDCs	CD68^−^, CD163^−^, CD11c^+^	++(+)
Follicular DCs	CD21^+^	−

Definition of abbreviations: Mac, macrophages; DCs, dendritic cells; mDCs, myeloid dendritic cells. IL-18 positive cells were identified in the lung of COPD patients (see Additional file 1: Table S2) by multi-color immunofluorescence staining using a panel of cell markers. There was no IL-18 staining associated with ECP^+^ eosinophils, MPO^+^ neutrophils, tryptase^+^ mast cells, CD3^+^ T cells, CD20^+^ B cells and CD138^+^ plasma cells. Interleukin-18 positivity was scored as follows: - absent staining, + weak light chromogen staining; ++, clear and moderate immunoreactivity; +++ intense dark staining (brackets are used to indicate where the higher scoring was seen only in certain patients and not all of those analysed)

### IL-1 and IL-18 differentially contribute to IFNγ induction in vitro

**Fig. 4:**
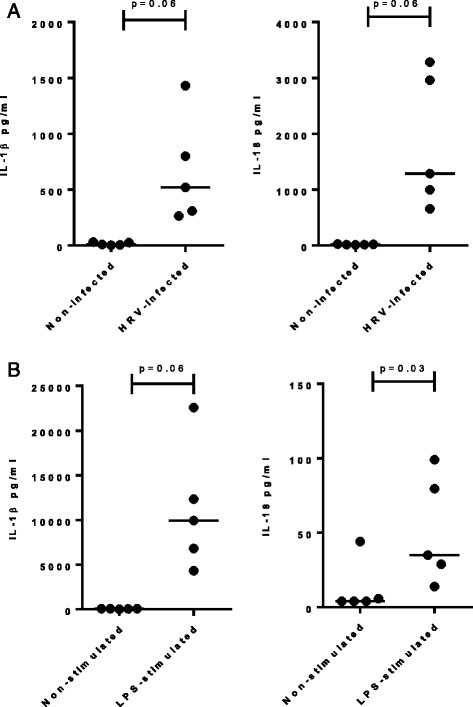
Induction of IL-1β and IL-18 release by NHBE cells infected with HRV and monocytes stimulated with LPS. IL-18 and IL1β production was measured by ELISA in the supernatant of NHBE cells 48 h after infection with HRV14 (**a**) and in the supernatant of human monocytes isolated from healthy donors and stimulated for 24 h with LPS (**b**). Data show individual data points for each donor and summary bars indicate median for each condition for *n* = 5 donors. The data were paired by donor and *p* values were calculated using a Wilcoxon signed-rank test

**Fig. 5:**
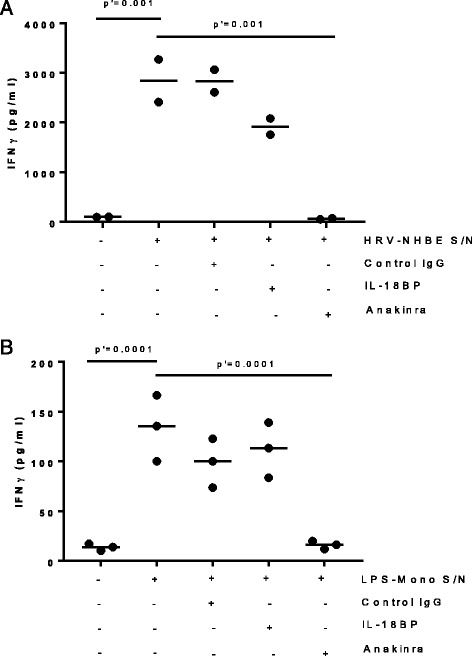
Blocking endogenous IL-18 activity did not significantly impact IFNγ release by NK cells stimulated with supernatants of infected NHBE cells or activated monocytes. Human NK cells were incubated for 24 h with supernatants from HRV14-infected NHBE cells (**a**) or LPS-treated monocytes (**b**) in the presence of IL-12, a known enhancer of IFNγ production. IFNγ production by NK cells was determined in the presence (+) of Anakinra (IL-1 antagonist), IL-18BP (IL-18 antagonist), a control IgG1 isotype or no addition (−). Due to donor to donor variations in the level of IFNγ response, the mean values across experiments were determined after normalization to IFNγ levels observed in the supernatant of NK cells stimulated in absence of antagonists. Data show the individual data points from independent experiments and the median (*n* = 2 (**a**) and *n* = 3 (**b**)). Statistical significance was assessed with the Friedman test with Dunn’s multiple comparison test (p’ indicates the adjusted *p* values)

T cells and NK cells, the likely source of IFNγ in the lung, are increased in numbers in COPD. To model what might be the primary downstream effect of IL-18 release in the lung in the context of other pro-inflammatory mediators, we studied the production of IFNγ by NK cells incubated with either lung epithelial cell- or monocyte-derived IL-18. As we have previously reported [19], infection of Normal Human Bronchial Epithelial (NHBE) cells with human rhinovirus resulted in the release of significant levels of IL-1β and IL-18 (Fig. 4a). These cytokines were also secreted by LPS-stimulated monocytes although the level of IL-18 was modest in comparison to infected NHBE cells (Fig. 4b). Human NK cells were incubated for 24 h with the supernatant of HRV-infected NHBE cells in the presence of IL-12, a known enhancer of IFNγ production. Neither IL-12 alone, nor the IL-1/IL-18-containing supernatant alone, were able to trigger IFNγ release from NK cells (data not shown, *n* > 4). Whilst IFNγ was detected when they were used in combination, this was not predominately driven by IL-18 as the addition of recombinant IL-18BP, a known IL-18 antagonist, had only a modest effect with a 32% reduction in IFNγ release (Fig. 5a). In addition, when NK cells were stimulated in the presence of IL-12 and supernatants from LPS-stimulated monocytes, IL-18BP did not significantly impact IFNγ production (Fig. 5b). IL-1R signalling has also been reported to trigger IFNγ production by NK cells [21]. Thus we used recombinant IL-1R antagonist (anakinra) to assess the contribution of IL-1α and IL-1β in our in vitro models of IFNγ induction. Addition of anakinra strongly inhibited IFNγ induction, irrespective of the use of HRV-stimulated NHBE supernatants or LPS-stimulated supernatants to activate NK cells (Fig. 5a-b).

**Fig. 6:**
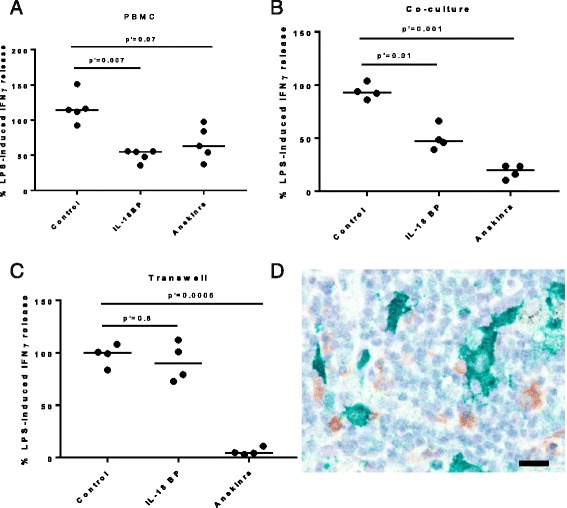
Induction of IFNγ by endogenous IL-18 required the IL-18-producing cells to be in close proximity to NK cells. IFNγ release was induced by stimulating either PBMC with IL-12 and LPS (**a**) or by stimulating NK cells with IL-12, LPS and monocytes in a co-culture system (**b**) or segregating NK and monocytes in a transwell system (**c**). IFNγ production by NK cells was determined in the presence of Anakinra (IL-1 antagonist), IL-18BP (IL-18 antagonist), or a control IgG1. Due to donor to donor variations in the level of IFNγ response, the mean values across experiments were determined after normalization to IFNγ levels observed in the supernatant of NK cells stimulated in absence of antagonists. Data show the individual data points and median from *n* = 5 donors (**a**) and *n* = 4 donors (**b**-**c**). p’ values calculated with Friedman test with Dunn’s multiple comparison test (**d**) Bright field micrograph exemplifying the typical and common close proximity of IL-18^+^ cells (Vina green chromogen) and CD56^+^ cells (brown DAB) in a lung section. Scale bar denotes 18 μm

This is in contrast to what could be observed when whole PBMC were stimulated with LPS and IL-12 in the presence of either IL-18BP or anakinra. In that context, both IL-18 and IL-1 contributed to IFNγ induction with a statistically significant increased contribution from IL-18 compared to NK cells cultured with supernatants alone (Fig. 6a). In an attempt to reconcile these observations, we compared the level of IFNγ release when NK cells were cultured with monocytes, either in the same well, or in a transwell system where they were prevented from coming into close proximity but able to exchange soluble mediators. The addition of anakinra resulted in a decrease in IFNγ production both in the co-culture and the transwell system, 82 and 94% respectively (Fig. 6b-c). By contrast, whilst IL-18BP did not have a statistically significant effect on IFNγ release in the transwell system (Fig. 6c), there was a 50% reduction when NK cells were co-cultured with monocytes (Fig. 6d).

Thus the downstream effect of physiological concentrations of endogenous IL-18 may depend on the spatial organization of IL-18-producing and responding cells. In order to confirm whether this close physical interaction could be observed in COPD lung, double immunostaining of COPD lung sections for IL-18 and CD56 indicated that in lymphoid aggregates in the lung, IL-18-positive myeloid cells are indeed frequently in close proximity to CD56^+^ lymphoid cells, either NK cells or subsets of CD8^+^ T cells (Fig. 6d).

## Discussion

IL-1 family members play an important role downstream of pattern recognition receptors in the maintenance of mucosal surface integrity. IL-1 and IL-18 in particular are associated with the recruitment and activation of myeloid and lymphoid effector cells. In this study, we have demonstrated that, in the context of lung inflammation involving a complex mix of mediators and despite disease heterogeneity, IL-1 and IL-18 are statistically significantly correlated with IFNγ in the sputum of COPD patients. Both mediators, released from epithelial cells or macrophages, likely are important in driving an IFNγ response in NK cells in the lung, with different spatial requirements. In agreement with recent observations in the human gastro-intestinal tract [22] we further revealed that IL-18^+^ macrophages and dendritic cells are in COPD foremost localized to lung lymphoid aggregates. Increased lymphoid aggregates in the lung are a histological hallmark of severe COPD status and this study is the first to highlight that IL-18- expressing myeloid cells within these lung lymphoid aggregates may significantly influence IFNγ release from closely-situated lymphocytes, such as NK cells, thereby contributing to tissue destruction in severe COPD pathology.

Interferon γ expression is expected within a pro-inflammatory lung environment and indeed, in 82 out of 105 COPD sputum from GOLD II and GOLD III patients the IFNγ level was detectable. Reports of the direct measurement of IFNγ at the protein level in the lung of COPD patients are scarce, most of the time relying on the isolation of NK or T cells from bronchoalveolar lavage or lung parenchyma followed by assessment of interferon response to stimulation ex-vivo [5, 23, 24], and where IFNγ levels have been measured in sputum, they are consistent with those reported here [12]. Interferon γ is a challenging cytokine to measure in sputum samples at least in part due to its relative sensitivity to sputum processing [25]. Dithiothreitol (DTT) used in standardized sputum processing could affect detection of specific soluble mediators, in addition to IFNγ, and although we did not systematically analyse the effects of DTT on our detection limits, it is likely that the presence of DTT could have uniformly affected select analytes across all samples. In addition, factors such as the severity of the disease, the triggers of exacerbation and the timing of sampling may influence the mediators that can be measured. We also note that sputum was obtained spontaneously, which confines the sputum analysis to a more chronic bronchitic patient phenotype, whereas the surgical samples used for IHC reflect a broader COPD population. In our sputum cohort, higher levels of IFNγ were associated with higher levels of IL-1α, IL-1β and IL-18, as well as general inflammation markers such as CRP and fibrinogen. Interleukin-18 receptor is primarily expressed on T cells, NK cells and subsets of innate lymphoid cells, and IL-18 is associated with the induction of IFNγ production by these cell types [26, 27]. Whilst IL-1 is generally linked to a neutrophilic response and Th17 cell differentiation, like IL-18, it has been reported to promote IFNγ release from cytotoxic lymphocytes [28, 29]. Although other inflammatory mediators may contribute to IFNγ production, we did not observe any correlation with IL-12, IL-15, or other molecules previously reported to be associated with IFNγ and/or viral infections such as IP-10, MIG, I-TAC, MCP-1, RANTES [12]. These observations re-inforce the likelihood of IL-18 and IL-1 being the strongest drivers of IFNγ.

In order to further understand the relationship between IL-18 and IFNγ in the lung tissue of COPD patients, we developed a protocol for the detailed phenotyping of IL-18-producing cells. Consistent with the previous observation that the bronchial epithelium is an important source of IL-18 following viral infection [19], we found IL-18 staining associated with bronchial epithelial cells. Given the rapid release of IL-18 following viral infection of NHBEs [19], it is of particular interest that we observed a novel and characteristic apical staining of IL-18 in airway epithelium in COPD, which is of stronger intensity than the general staining in epithelium, suggesting IL-18 is concentrated at the apical surface of differentiated epithelium, possibly localized for rapid release. Given that the extent of this apical staining correlated with disease severity, further studies to evaluate the contribution of IL-18 in viral exacerbations in COPD patients are warranted. We have not explored here whether IL-18 released from the epithelium can activate lymphocytes in the airway lumen, or whether the IFNγ measured in sputum is purely as a result of cytokine production within the tissue. This could warrant further analysis since we suggest locally high production of IFNγ within the tissue is more likely to invoke detrimental effects to tissue structure, whilst epithelial release of IL-18 may be beneficial to pathogen clearance.

In addition to moderate alveolar macrophage expression, we saw very strong staining in macrophages and dendritic cells associated with lymphoid aggregates in the lung, which was not seen for IL-1 expression, despite observing IL-1^+^ macrophages in the tissue [17]. We demonstrated that the overall expression of IL-18 in the lung increased as a direct consequence of the enhanced number of macrophage and dendritic cell-enriched alveolar-lymphoid interfaces in severe disease. It has been demonstrated that not only bronchiolar associated lymphoid structures, but also alveolar aggregates, correlate with disease severity and may represent remodelled antigen presentation in the COPD lung [3]. This is the first description of IL-18 expression associated with myeloid cells specifically in tertiary lymphoid structures in COPD, and was not reported in a previous study of IL-18 staining in COPD lung [18]. We note that the staining in lymphoid aggregates is strikingly similar to IL-18 staining reported by Bombadieri et al. in tertiary follicles found in salivary glands in Sjörgren’s Syndrome [30], and the presence of IL-18 producing cells in lymphoid aggregates in Crohn’s disease [22]. Given elastin autoreactive T cells isolated from COPD blood have been shown to produce significant quantities of IFNγ [31] and lymphoid follicles in more severe COPD have been linked to an autoimmune-like phenotype (reviewed in [32]) this is a particularly striking parallel.

Given the clear association of IL-18 with lymphoid aggregates, with a pattern of distribution of IL-18^+^ cells which closely represents that described in lymph nodes in infection (including sub-capsular like IL-18^+^ cells) [8], we hypothesised that the same mechanisms which relate to cellular positioning described in lymph nodes to regulate IFNγ may be important in these ectopic follicle-like structures in the lung, and therefore become increasingly dominant as COPD progresses. However, quantifying IFNγ expression by IHC or ISH in tissues is technically challenging and we were not able to perform this analysis to our satisfaction in formalin-fixed paraffin embedded COPD lung tissues that were available for this study. Kastenmuller et al. [8] described how myeloid cells, acting as sentinels in the earliest detection of pathogens in the lymph node, were closely localised with diverse lymphoid cells in order to rapidly facilitate an innate immune amplification loop to minimise systemic pathogen spread. Their data suggested that IL-18 with a second signal was responsible for a rapid increase in lymphoid cell IFNγ production driven by cellular proximity. We therefore tried to understand the relationship between endogenous IL-18 release and IFNγ response in pathologically relevant primary cell in vitro model systems. Whilst the contribution to NK IFNγ production by IL-1 was immediately apparent, the dependence on IL-18 for this response was crucially only revealed when IL-18-producing cells were cultured in close proximity to the IFNγ-producing cells. Co-staining for IL-18 and CD56^+^ in COPD tissue, the close association of CD56^+^ NK/NKT cells with IL-18^+^ cells could be observed in the lymphoid aggregates, reminiscent of what has been reported in secondary lymphoid organs.

Although less frequent in control samples, or mild disease, lymphoid aggregates are occasionally observed, and this is likely as a normal response to a previous infection. The increase in lymphoid aggregates and concomitant increase in IL-18 may therefore be a physiological response to increased bacterial colonisation and bacterial and viral infection frequency in the vulnerable lungs of severe COPD patients. However, they may also contribute to emphysema progression, particularly since the localised effects of IL-18 on tissue resident responder cells may be to increase IFNγ without the requirement for cognate antigen. Both IL-18 and IFNγ have been reported to contribute to emphysema pathogenesis in mouse smoke [15, 16, 33, 34], and increases in lymphoid aggregates are reported in mouse smoke models in a similar timeframe to emphysema progression [35]. IL-18 has been shown to stimulate increased IFNγ production by lymphocytes isolated from COPD lung compared to smoking control tissue [5], and the cytotoxic potential of CD56^+^ cells increased with disease severity [6], therefore future studies should attempt to determine whether this phenotype is indeed evident within lymphoid follicles in COPD lung tissue with IL-18 as a key driver. Recent data also showed that CD4^+^ cells expressing IL-18Rα at mucosal surfaces have innate immune functionality and co-localise with IL-18-producing cells in lymphoid aggregates in chronic inflammation in the gut [22]. Additionally, Kearley et al. have described an altered phenotype and responsiveness of NK and ILC1s in smoked mice, which could also be relevant to plasticity of the innate cell responders and production of IFNγ in COPD [36].

## Conclusions

We have shown that IL-18 released in pathologically relevant situations, such as from epithelium after infection or from myeloid cells in lymphoid aggregates, has a spatially limited function with respect to its rapid innate regulation of IFNγ. In contrast to its physiological function to control infection spread in lymphoid organs which is tightly regulated, this local interface between IL-18 expressing cell and responder cell is increasingly supported in the lung tissue as the severity of disease increases, and this may directly impact its potential to contribute to tissue damage via augmented IFNγ release. This may have been underappreciated in previous studies of COPD if the localisation of IL-18 and its functions has not been taken into account, and may also be relevant in other chronic inflammatory diseases.

## Additional files

Additional file 1: Supplementary Tables. (DOCX 26 kb)

Additional file 2: Supplementary Figures. (PDF 696 kb)

## Abbreviations

(F)VC: (Forced) vital capacity
COPD: Chronic obstructive pulmonary disease
FEV_1_: Forced expiratory volume in one second
GOLD: Global initiative for chronic obstructive lung disease
HRV(14): Human rhinovirus (strain14)
LA: Lymphoid aggregates
LDD: Least detectable dose
LPS: Lipopolysaccharide
NHBE: Normal human bronchial epithelial cells
PBMC: Peripheral blood mononuclear cells
