# Supplementary material for: IL-18 associated with lung lymphoid aggregates drives IFNγ production in severe COPD
Source: Respir Res. 2017 Aug 22;18:159. doi: 10.1186/s12931-017-0641-7 (PMC5568255; doi:10.1186/s12931-017-0641-7)
Supplement: Supplementary file 2 — Supplementary Figures. (PDF 696 kb) [file 12931_2017_641_MOESM2_ESM.pdf]

IL-18 associated with lung lymphoid aggregates drives IFNγ production in severe COPD

Emmanuel Briend [a,1], John G. Ferguson [a], Michiko Mori [c], Gautam Damera [b], Katherine Stephenson [a,2], Natasha A.Karp[f], Sanjay Sethi [e], Christine K. Ward [b,3], Matthew A. Sleeman [a,4], Jonas S. Erjefält [c,d], and Donna K. Finch [a,5]

[a]MedImmune Ltd, Granta Park, Cambridge CB21 6GH, United Kingdom

[b]MedImmune LLC, 1 MedImmune Way, Gaithersburg, MD, USA

[c]Department of Experimental Medical Science, BMC D12, Lund University, SE-221 84, Lund, Sweden

[d]Department of Respiratory Medicine and Allergology, Lund University Hospital, Lund, Sweden

[e]Department of Medicine, University at Buffalo, 3495 Bailey Avenue, Buffalo NY 14215, USA

[f]Quantitative Biology IMED, AstraZeneca R&D, Cambridge, UK

[1]Current affliation: Agenus Ltd, Cambridge, UK

[2]Current affliation: University of Nottingham, UK

[3]Current affliation: Bristol-Myers Squibb, Princeton, NJ, USA

[4]Current affliation: Regeneron Pharmaceuticals Inc, Tarrytown, NY, USA

[5]Corresponding author:

Donna Finch PhD,

MedImmune Ltd,

Granta Park,

Cambridge CB21 6GH, United Kingdom

Tel: +44 (0)1223 471471

Fax: +44 (0)1223 471472

Finchd@medimmune.com

Supplementary figures

Supplementary Figure 1

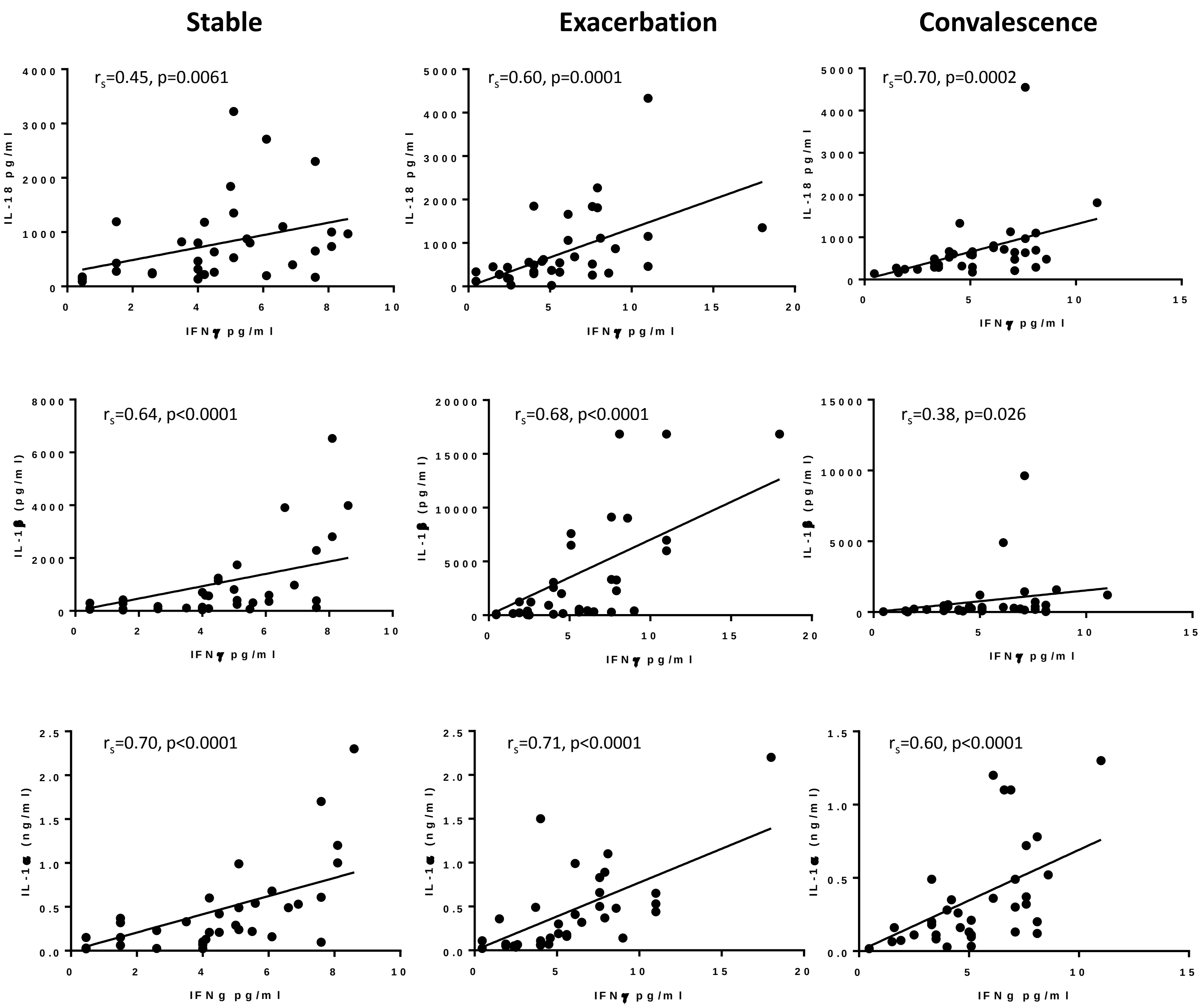

Supplementary Figure 2

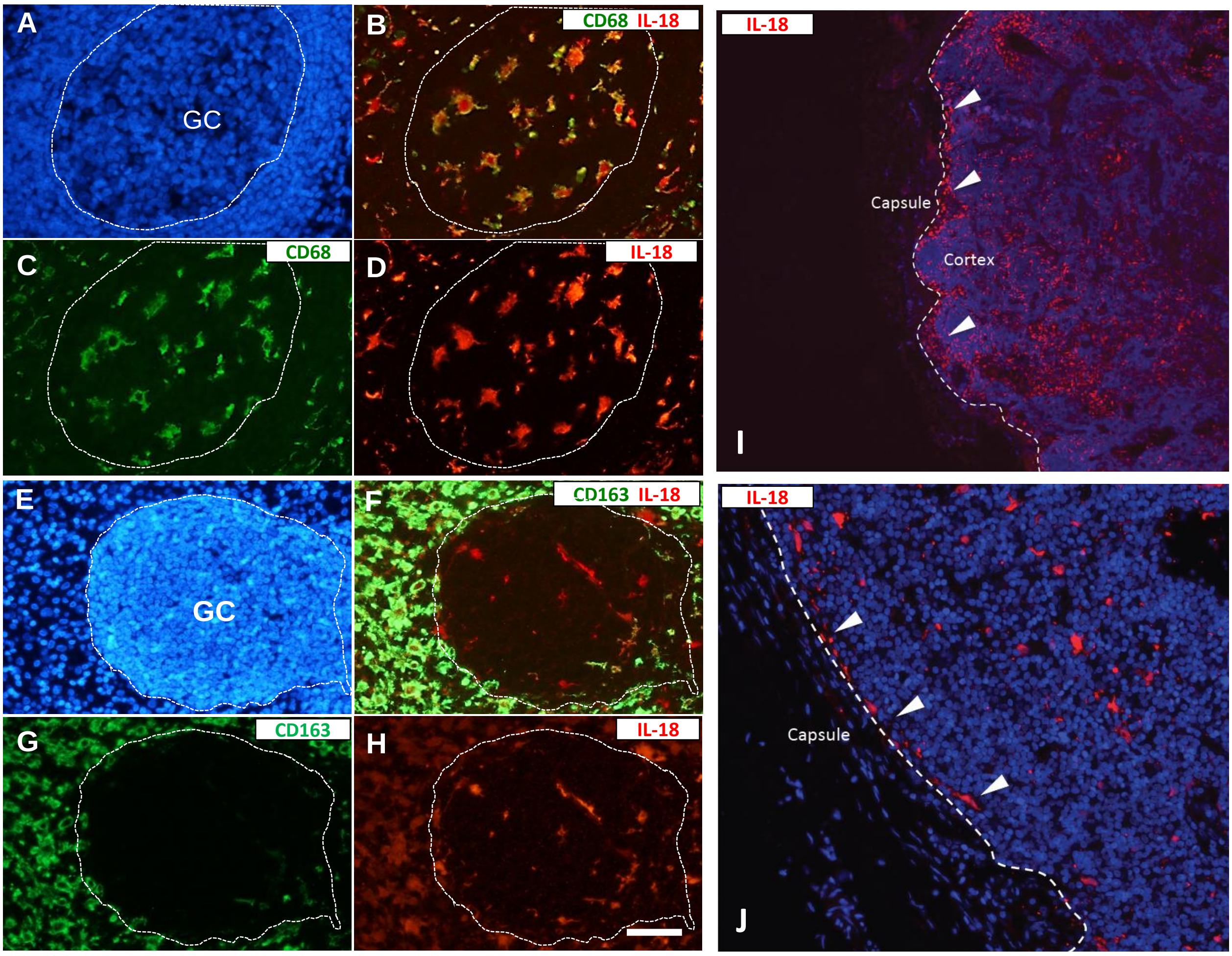

(A-H) Immunofluorescence staining of lung draining lymph nodes identifying IL-18 expressing cells based on the markers CD68 and CD163. Cell nuclei were stained with Hoechst 33342 (A, E, blue). Cells were stained for CD68 (C, green), CD163 (G, green) and IL-18 (D, H, red). The overlay of IL-18 staining with either CD68 or CD163 is shown in (B) and (F) respectively. Most CD68+ cells within the germinal center or in the T cell area were IL-18+. Some IL-18+ CD163+ cells were observed outside the germinal center.

(I-J) Immunofluorescence staining of IL-18 (red) in lung draining lymph nodes exemplifying the presence of IL-18 positive macrophage-like cells in the subcapsular region (arrows) as well as in the cortex.
