# Supplementary material for: IL-18 associated with lung lymphoid aggregates drives IFNγ production in severe COPD
Source: Respir Res. 2017 Aug 22;18:159. doi: 10.1186/s12931-017-0641-7 (PMC5568255; doi:10.1186/s12931-017-0641-7)
Supplement: Supplementary file 1 — Supplementary Tables. (DOCX 26 kb) [file 12931_2017_641_MOESM1_ESM.docx]

July 26, 2017

Respiratory Research Editorial Office

RERE-D-17-00079

Re: IL-18 associated with lung lymphoid aggregates drives IFNγ production in severe COPD
Emmanuel Briend; John Ferguson; Michiko Mori; Gautum Damera; Katherine Stephenson; Sanjay Sethi; Christine Ward; Matthew Sleeman; Jonas Erjefalt; Donna K Finch

Dear Professor Ammit,

Please accept our thanks for your editorial assessment, and to the reviewers for their expert review of our manuscript ‘IL-18 associated with lung lymphoid aggregates drives IFNγ production in severe COPD’ (RERE-D-17-00276). Please find our responses to the Reviewer comments below, and our updated manuscript, figures and supplementary materials also attached.

We found the reviewers comments to be very helpful and we believe we are submitting a significantly improved manuscript, particularly with respect to statistical analyses and interpretation which was emphasized by Reviewer 1. We re-analyzed and re-plotted much of the data, and edited the manuscript in this respect, with the expert help of statistician, Natasha Karp, and we would therefore like to add her to the new author list. We have submitted a completed new authorship request form to BioMed Central with the revised manuscript signed by all authors, original and added.

Due to some issues with the original receipt of the comments from Reviewer 1, we have included our responses below in this letter to their comments in full (as received by email on 6 April 2017), as well as in the automated form.

**Responses to reviewers:**

Reviewer reports:

*Reviewer #1: Given my questions regarding statistical analyses below, I recommend that this paper be reviewed by a statistical reviewer.*

See point by point response below.

*Reviewer 1 comments received by email from Editorial office:*
*Briend and colleagues report on the increase of IL-18 in the sputum of COPD patients compared to controls and how this appears to associate with IL-18 expression by lung epithelial cells. This epithelial expression could be mimicked in vitro by stimulation of NHBE by HRV infection. They go on to demonstrate that there is also strong IL-18 expression by myeloid cells in the lymphoid tissue and speculate that this IL-18 expression is involved in IFNgamma release by NK cells and T cells in the lymphoid tissue. They go on to test this assumption via the use of an IL-18 antagonist and transwell experiments and demonstrate that cell contact seems to be required to mediate the effect of IL-18 on IFNgamma production by NK cells.*

*Major comments:*

1. *Whilst I am not a statistician, I question some of the statistical tests performed. For example, in Fig 1A samples were from the same patient and could therefore be considered as paired so perhaps a Friedman ANOVA would be more appropriate. A similar comment could be made about Figure 6. Moreover, if data is considered nonparametric, as in the case of Fig 1B which has been analysed by Mann Whitney, then it would be more appropriate to present the data as median bars rather than mean+/- SD. In Fig 4, a t test is used when a Wilcoxon test appears to be most appropriate. My biggest statistical concern is that of the 175 analytes tested in the sputum, 8 of them could be higher as a result of chance (if p<0.05 is used). As there are so many analytes, would it not be better to perform some sort of FDR correction similar to those used in microarray analyses?*

We have reviewed all and revised many of the statistical analyses of the data in our manuscript, making use of a linear mixed effect model for the sputum analyses at stable, exacerbation and convalescence status, for example. We have amended analysis to take into account assessments of distribution characteristics of the data, to adjust for multiple testing and to account for structure in the data such as data being paired by donor. Where required we have revised the wording in methods, results and figure legends to ensure statistical robust representation of the data in the manuscript. We did not find our conclusions were altered by these analyses, however we are satisfied the statistical robustness of the paper is much improved by these alterations.

*2.	In terms of the sputum analysis, DTT can have large effects on the ability to measure many cytokine analytes. Did the authors perform some validation or spiking experiments to ensure that there was no effect of the matrix on their ability to measure specific analytes? Can the multiplex assay used differentiate between pro- and mature forms of the cytokines of interest? I also find the description of the samples used a little confusing in both the methods section and table/figure legends. If 24 patients were included, were 35 exacerbation samples and 35 convalescent samples compared to 24 baseline/screening samples? How have the authors dealt with possible confounding as a result of dealing with possible repeated measures from the same individuals? It is possible that restricting analysis to the first exacerbation (and associated convalescent sample) could reduce the risk of confounding. The sputum analysed was obtained spontaneously, which would suggest that the COPD cohort were more of a chronic bronchitic phenotype than emphysematous and should be discussed as the study results may therefore not apply to all COPD phenotypes.*

Although some sputum spiking experiments have been carried out by the supplier (Rules Based Medicine) we did not robustly test the effect of DTT in the sputum analysis, therefore we have added a discussion of this point to our Discussion section to capture this caveat. We did not assess the ability of the RBM assay to detect pro- or mature forms of IL-18 or IL-1beta, although we do assess functional activity of endogenously released proteins in our in vitro assays and these would contain mature active cytokine since neither pro-IL-18 nor pro-IL-1beta is considered to be active at their respective receptors. We previously studied pro- and mature forms of IL-1 family members during HRV-induced release from epithelial cells in more detail (Piper et al 2013, PlosOne <https://doi.org/10.1371/journal.pone.0063365>). We consider further study of processing of IL-18 and IL-1beta in the diseased tissue to be out of the scope of the current manuscript, although certainly an interesting point to consider for future studies. We have changed the description of the samples in methods to make it clear that some of the patients had more than one exacerbation event. We have re-analysed the data according to event status using a linear mixed effect model to account for repeated measures from a patient and will appropriately separate the effect of patient variation from the impact of disease. This is now described in detail in the Methods. We have added a statement to the discussion to acknowledge the potential sample bias in the sputum analysis due to the use of spontaneous sputum, whilst stating the IHC cohort were more randomly sampled.

*3.	I do not find Table 2 to be at all helpful to the authors argument. It may perhaps be better to simply state that the cytokines correlate irrespective of status and remove the statement regarding the marginally higher rank r value. By contrast in Figure 3, it would be helpful to have the r and p value indicated on the graph. The data in Figure 5 are based on a n=2 and n=3 and so I am unsure as to how robust the statistical tests performed are.*

We have removed Table 2, replacing with a Supplementary figure which shows the correlations graphically. We have added the r_s_ and p values to Figure 3. We have re-graphed and reanalysed the data in Figure 5, and while the n numbers are small the effect size is large. We have recognised that there is connection in the data by donor, and we have analysed the data accordingly.

*4.	There appears to be a disconnect between the luminal measurement of IL-18 in sputum and the epithelium and that in the lymphoid tissue as the authors do not present any data on lymphocytes in the lumen. Have these been quantified in the sputum samples analysed and do they correlate with IL-18 production? Some discussion of the possible different roles of myeloid and epithelial expression of IL-18 is warranted.*

This is a very interesting point, and we have recognised this in the Discussion. There may indeed be a benefit in luminal release of IL-18 to increase clearance of virus, versus increased tissue damage when IL-18 is released locally at high concentrations in the tissue. We are not able to address this in this study, as we received processed sputum, and could not therefore isolate or analyse the responses of lymphocytes from the airways.

*Minor points:*

1. *The figure legend of supplementary figure 1 describes CD63 rather than CD68*

Amended

2. *Figure 6 is based on stimulation of cells with both LPS and IL-12 yet on LPS is indicated, IL-12 needs to be indicated in the figure to avoid confusion. Furthermore, the figures are not presented in a very logical order.*

We have re-drawn and re-ordered Figure 6. Since we normalise to the LPS + IL-12 response, including these points in the original graph do not add value (because all of the LPS and IL12 alone will be 100%), and this also is statistically incorrect to include this group in an ANOVA because by doing so we remove variation in the original comparator arm (LPS + IL12 alone). Therefore, we now present the data of the control Fc group, anakinra and IL-18BP only as % normalised IFNγ, and describe in the method, results and legend that these data were normalised according to the maximal stimulation with LPS and IL-12. The statistical analysis was then performed comparing anakinra or IL-18BP to control IgG1.

*3. Why look at virus in the epithelium and LPS in PBMC? Why not concentrate on one stimulus as monocyte/macrophages can be virally infected and similarly both cell types respond to LPS?”*

We feel the data are stronger by addressing both virally induced and LPS induced endogenous IL-18/IL-1 release. Whilst HRV can infect alveolar macrophages from tissue digests, we were not convinced this would be physiologically relevant for the IL-18-positive myeloid cells in the lymphoid aggregates, which are not surveying the epithelium in the same way as alveolar macrophages, although we acknowledge we are using monocyte derived cells for the co-culture and transwell experiments for practical reasons.

*Reviewer #2: The authors identified the upstream mediators of IFN-γ in CODP patients, and further clarified the source of IL-18. The findings indicated the importance of local interface between IL-18-expressing and responder cell in disease progress. The study is well designed and the results support the conclusions.*
*Minor comments:
1) In Figure 1, it would be better to add the control groups (eg. Never smokers and smokers without COPD) as well.*

At the time these analyses were run for the COPD sputum, we did not have sputum from these control groups as these were not collected under this protocol, rather we compared stable with exacerbation state in a longitudinal study. We did collect samples at a later date from control smokers and never smoker volunteers and detected IL-18 and IL-1-α and -β far less frequently in these samples. However the RBM assay had been re-developed by the supplier in the meantime using different detection antibodies to our initial study and these control data are therefore not appropriate comparator groups for the COPD sputum data in this manuscript.

We hope that you can accept this manuscript for publication in your journal following our responses to the reviewer comments above.

Best Regards,

**Donna Finch PhD**

**Principal Scientist**

**Respiratory, Inflammation and AutoImmunity**

**MedImmune Ltd**

Milstein Building

Granta Park

Cambridge

CB21 6GH

Direct line: +44 (0)203 749 6214

Mobile: +44 (0)7917 810828

[finchd@medimmune.com](mailto:finchd@medimmune.com)

[www.medimmune.com](http://www.medimmune.com/)
